# Effect of Low-Intensity Endurance Training and High-Intensity Interval Training on Sperm Quality in Male Rats with Fatty Liver

**DOI:** 10.22074/IJFS.2020.134593

**Published:** 2021-03-11

**Authors:** Mahnaz Hosseini, Seyyed Vajiheh Alsadat Hashemi, Mohammad Hossein Bagheri, Marziyeh Tavalaee, Seyed Morteza Seifati, Dina Zohrabi, Mohammad Hossein Nasr-Esfahani

**Affiliations:** 1 Department of Animal Biotechnology, Reproductive Biomedicine Research Center, Royan Institute for Biotechnology, ACECR, Isfahan, Iran; 2 Department of Biology, Faculty of Science, Nour Danesh Institute of Higher Education, Isfahan, Iran; 3 Biology Department, Medical Biotechnology Research Center, Ashkezar Branch, Islamic Azad University, Ashkezar, Yazd, Iran; 4 Department of Exercise Physiology, Faculty of Sport Sciences, Shahrekord University, Shahrekord, Iran

**Keywords:** DNA Damage, Fatty Liver, High-Intensity Interval Training, Oxidative Stress, Sperm

## Abstract

**Background:**

We aimed to investigate the effect of low-intensity endurance training (LIET) and high-intensity inter- val training (HIIT) on sperm parameters, chromatin status, and oxidative stress in a rat model of non-alcoholic fatty liver disease (NAFLD).

**Materials and Methods:**

For this experimental study, we divided 40 male Wistar rats into four groups (control, sham, HIIT and LIET) according to diet treatment and exercise training protocol. Liver triglycerides, sperm parameters, sperm lipid peroxidation (BODIPY C11 probe) and chromatin status [chromomycin A3 (CMA3)], and acridine orange [AO] staining) were assessed in these groups at the end of the study.

**Results:**

The mean liver triglyceride values significantly improved in both the LIET and HIIT groups compared to the control and sham groups. The mean of testicular volume, sperm concentration, motility, intensity of sperm lipid peroxidation and DNA damage were similar within groups. While, the mean percentage of sperm lipid peroxidation and protamine deficiency were significantly higher in the LIET and HIIT groups compared to the control group.

**Conclusion:**

Both LIET and HIIT in the rat NAFLD model had no adverse effects on testicular morphometric param- eters, sperm concentration, motility, and DNA integrity. However, the mean sperm lipid peroxidation and protamine deficiency were significantly higher in both exercise groups. Our study suggests that exercise or antioxidant supple- mentation could minimise the adverse effects of oxidant by-products of exercise.

## Introduction

Overweight and obesity are medical conditions defined as excessive amounts of fat in the body. Both have led to increases in health-related issues in developed countries. Obesity increases the risk for diabetes, high blood pressure, heart disease, fatty liver and certain cancers. The results of numerous studies have shown a multifaceted relationship between obesity and low sperm quality and male infertility (1). In addition, obesity could lead to an increased time to pregnancy, reduced pregnancy rate, and loss of pregnancy in couples who undergo assisted reproductive technology (2). More importantly, Li et al. (3) demonstrated offspring of obese fathers were more likely to be at an increased risk for obesity. Therefore, weight reduction could possibly improve the health of the next generation. It has been reported that obesity is one of the most important reasons for liver steatosis, an accumulation of fat in the liver (4). Imbalances and disturbances in any of the metabolic pathways can affect liver function and ultimately lead to non-alcoholic fatty liver disease (NAFLD). Different degrees of NAFLD are attributed to impairments of lipid synthesis mechanisms and oxidation pathways. Cell damage, oxidation of fatty acids in mitochondria, and prevention of triglyceride outflow all contribute to steatosis in NAFLD (5). The combination of diet and physical activity are the most effective NAFLD treatment strategies (6). Lifestyle changes can lead to reductions in abdominal fat, blood lipid levels and intracellular liver contents, which directly reduce glucose production in the liver and improve insulin sensitivity (7). Exercise is a key component of weight management and may also play an important role in preventing infertility.

Studies have shown that the combination of an appropriate diet and exercise in obese mice improved the basic parameters of sperm that included motility, morphology, DNA fragmentation and mitochondrial reactive oxygen species (ROS) (8). Moderate exercise can be beneficial for fertility, but there is also evidence that intensive physical activity, such as professional biking, can have a damaging effect on fertility (9). Exercise intensity may have a destructive effect on hormonal and seminal fluid content, and may lead to oxidative conditions (10). Sports protocols, depending on the exercise method or intensity of physical activity, may have a positive or negative effect on semen quality. Data show that exercise and regular physical activity improve semen quality parameters and sperm DNA quality in both fertile and infertile populations. This effect may be due to the anti-inflammatory and antioxidant effects of exercise (11, 12).

Evidence suggests that resistance exercise is associated with oxidative stress. During rest, the human body continuously produces ROS; however, in healthy people, ROS is produced at levels that are within the capacity of the antioxidant system of the body. During resistance exercise, there is an increase in body oxygen consumption of 10-20 times (13), and the intake of oxygen in active skeletal muscle increases 200-100 fold (14). This increase in oxygen consumption can lead to overproduction of ROS, which exceeds the body's detoxification capacity (15). However, in some studies, an association between exercise and oxidative stress was not observed (16). Additional studies are required to confirm the effectiveness of exercise on sperm quality and fertility potential. This study, for the first time, aimed to assess the effects of both low-intensity endurance training (LIET) and high-intensity interval training (HIIT) on testicular morphometric, sperm parameters, oxidative stress and chromatin integrity in a rat model of NAFLD.

## Materials and Methods

### Ethical consideration

The Sport Science Research Institute of Iran (Trace Code 48010) approved this study. The research was carried out at Royan Institute (Isfahan, Iran). All the experiments were conducted in accordance with guidance from the Ethical Committee for Research on Laboratory Animals (Code: IR.SSRI.REC.1397.331).

### Animals

**Fig.1:**
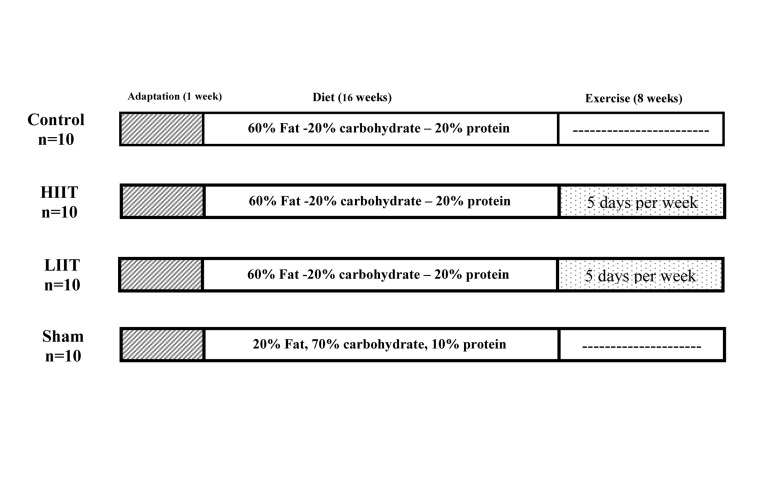
Study design, diet, and time of exercise protocol within the control, sham, HIIT, and LIIT groups. HIIT; High-intensity interval training and LIET; Lowintensity endurance training.

For this study, 40 male Wistar rats (5 weeks old) were used. The rats were housed in a room with five rats per cage, and allowed to acclimatise to a controlled 12-hour light/12- hour dark schedule and room temperature of 20-23°C. Food and water were provided ad libitum. After one week in this environment, we randomly divided the rats into four groups according to their diet treatment for 16 weeks and exercise training protocol for 8 weeks [HIIT and LIET, Fig.1, Table 1 (17-19)]. The rats had a mean weight of 156.4 ± 21.7 grams (150-200 grams) at the beginning of the study. The sham group had a diet comprised of 20 fat, 70% carbohydrates, and 10% protein, whereas the diets for the control, HIIT, and LIET groups were similar and consisted of 60 fat, 20% carbohydrates, and 20% protein. The control group did not undergo any training, whereas the HIIT and LIET groups underwent their respective training protocols. 

### Testicular evaluation and epididymal sperm extraction

We assessed testicular weight (g) and volume (ml) in the left and right testes of the rats. The epididymides were dissected from the testes, and the testes were washed and fixed in Bouin fixative to evaluate the seminiferous tube status of each testis. The blood vessels were removed from the epididymides, and we separated the caudal epididymides from the other parts of the epididymides, minced them, and placed them in a petri dish that contained 3 ml of VitaSperm and 10% foetal serum albumin (Inoclon, Iran) for 20 minutes. The released sperm were used to assess the study parameters.

**Table 1:** Patients’ characteristics in the successful and unsuccessful delivery groups


Week	Groups	Number of practice sessions per week (day)	Intensity during exercise (max) (%)	Intensity at rest (max)	Speed(m/minutes)	Number of exercise fields (HIIT)	Total time of LIET (minutes)	Total distance travelled in each session (work and rest) (meters)

1	HIIT	5	75	30	30	2 repetitions	------	246
	LIET	5	45	-----	18	-----	17	246
2	HIIT	5	80	30	32	4 repetitions	-------	445
	LIET	5	45	-------	18	-------	28	445
3	HIIT	5	85	30	34	6 repetitions	-------	588
	LIET	5	45	-------	18	-------	36	588
4	HIIT	5	90	20	36	8 repetitions	-------	748
	LIET	5	45	-------	18	-------	45	748
5	HIIT	5	90	20	36	8 repetitions	-------	748
	LIET	5	45	-------	18	-------	45	748
6	HIIT	5	90	20	36	8 repetitions	-------	748
	LIET	5	45	-------	18	------	45	748
7	HIIT	5	90	20	36	8 repetitions	-------	748
	LIET	5	45	-------	18	-------	45	748
8	HIIT	5	90	20	36	8 repetitions	-------	748
	LIET	5	45	-------	18	-------	45	748
1	HIIT	5	75	30	30	2 repetitions	------	246
	LIET	5	45	-----	18	-----	17	246
2	HIIT	5	80	30	32	4 repetitions	-------	445
	LIET	5	45	-------	18	-------	28	445
3	HIIT	5	85	30	34	6 repetitions	-------	588
	LIET	5	45	-------	18	-------	36	588
4	HIIT	5	90	20	36	8 repetitions	-------	748
	LIET	5	45	-------	18	-------	45	748
5	HIIT	5	90	20	36	8 repetitions	-------	748
	LIET	5	45	-------	18	-------	45	748
6	HIIT	5	90	20	36	8 repetitions	-------	748
	LIET	5	45	-------	18	------	45	748
7	HIIT	5	90	20	36	8 repetitions	-------	748
	LIET	5	45	-------	18	-------	45	748
8	HIIT	5	90	20	36	8 repetitions	-------	748
	LIET	5	45	-------	18	-------	45	748


LIET; Low-intensity endurance training and HIIT; High-intensity interval training.

### Sperm parameters

We assessed sperm motility by placing 10 µl of the sperm sample obtained from the cauda epididymides on a slide. Motility was observed by using an optical microscope (CX31 Olympus, Dubai, UAE). For each animal, at least 200 sperm cells were evaluated from different fields and the mean percentage of motile sperm was recorded. Sperm concentrations were evaluated using a sperm counting chamber (Sperm meter, Sperm Processor Pvt. Ltd., Garkheda, India) and a Labomed CxL optical microscope (magnification: ×20). The results were reported and 10^6^ per ml. For sperm morphology, 30 µl of the sperm recovered from the cauda were stained with eosine/nigrosine according to a study of Afiyani et al. (21). 

### Sperm lipid peroxidation and chromatin status

Lipid peroxidation, protamine deficiency, and DNA damage in the sperm were assessed with a BODIPY C11 probe, and chromomycin A3 [CMA3] (22), and acridine orange (AO) staining, respectively, according to Afiyani et al. (21) with minor modifications. For assessment of sperm lipid peroxidation, we added BODIPY stain to 2×10^6^ washed epididymal sperm for a final concentration of 5 mM BODIPY/dimethyl sulfoxide (DMSO) in the presence (positive tube) and absence (test tube) of H_2_O_2_ for 30 minutes in the dark. Then, the samples were washed with phosphate-buffered saline (PBS) buffer at 500 g for 5 minutes. We calculated the percentage and intensity of sperm lipid peroxidation with a FACSCalibur flow cytometer (Becton Dickinson, San Jose, CA, USA).

For assessment of sperm protamine deficiency, the epididymal washed sperm were fixed with Carnoy’s solution (3:1 ratio of methanol: acetic acid, 3:1) for 5 minutes at -4 °C. Then, for each sample, we prepared two smears and the slides were stained with 100 µl of CMA3 solution (0.25 mg/ml). After one hour, the slides were washed with a PBS solution and air-dried. Then, the slides were covered with a coverslip and we counted 200 spermatozoa on each slide using an Olympus fluorescent microscope (BX51, Japan) with the appropriate filters (460-470 nm). Sperm that had a bright yellow stain were considered to be protamine-deficient (CMA3 positive spermatozoa), while sperm that had a dark yellow stain were considered to have normal protamine content (CMA3 negative spermatozoa). Finally, we reported the percentage of sperm that were protamine deficient for each sample.

Damage to the sperm DNA was assessed as follows. Aliquots of 20 µl of cauda-retrieved sperm cells were smeared onto the slides and fixed overnight with Carnoy’s solution at 4°C. Then, the slides were stained using 150 µl of a freshly prepared AO solution (in 0.1 M citric acid, 0.3 M NaH_2_PO_4_, pH=2.5) in the dark at room temperature. The slides were washed twice with PBS and observed with an Olympus fluorescent microscope (BX51, Japan) and the appropriate filters (460-470 nm). We counted 200 sperm cells on each slide and calculated the percentage of DNA damaged cells (cells that had a red/orange nucleus).

### Statistical analysis

We analysed the data with the Statistical Package for the Social Sciences (version 23.0, Chicago, IL, USA). The Shapiro-Wilk test was used to assess for normality of the distribution. One-way analysis of variance (ANOVA) was used to compare the parameters within the study groups. Data in the text and figures are presented as mean ± standard deviation of the mean (SDM). P<0.05 was considered to be statistically significant. 

## Results

### Animal weight, serum alanine transaminase and triglyceride levels

The rats had the following mean weights: 414.8 ± 4.2 g (sham), 452.5 ± 8.7 g (control), 388.00 ± 9.37 g (LIET), and 405.5 ± 10.09 g (HIIT) at the end of the study. We observed a significant reduction in weight in the LIET and HIIT groups compared to the control group (P<0.05). The control group had a significantly higher mean weight compared to the sham group (P=0.01).

This study was a continuation of a study by Bagheri et al. (20). In order to confirm the presence of fatty liver disease in the control, HIIT, and LIET groups, we used the GOT/ALT kit (Pars Azmun, Iran) and spectrophotometry to assess alanine transaminase (ALT) levels after 16 weeks. The mean ALT levels in the fatty liver groups (control, HIIT, and LIET) was significantly higher than the standard diet group (sham) (105.1 ± 9.6 vs. 60.7 ± 5.5 U/L). After confirmation of NAFLD, the HIIT and LIET groups began their exercise training protocols. At the end of the study, the level of liver triglycerides was assessed by the GPO-PAP kit (Pars Azmun, Iran) and spectrophotometry. There was a significant reduction in this parameter in both the HIIT (184.34 ± 28.69) and LIET (246.22 ± 35.94) groups compared to the control group (328.2 ± 27.74) (P<0.05).

### Macroscopic findings

We evaluated testicular weight (g) and volume (ml) the left and right testicles in the study groups. The mean testicular weights in the control, sham, LIET, and HIIT groups were not significantly different between the right (1.45 ± 0.05 g [control], 1.38 ± 0.06 g [sham], 1.28 ± 0.11 g [LIET] and 1.15 ± 0.19 g [HIIT]) and left (1.43 ± 0.05 g [control], 1.41 ± 0.04 g [sham], 1.32 ± 0.12 g [LIET] and 1.19 ± 0.19 g [HIIT]) testes. There were no significant differences in mean testis volume between the right (1.45 ± 0.05 ml [control], 1.45 ± 0.09 ml [sham], 1.24 ± 0.13 ml [LIET] and 1.1 ± 0.18 ml [HIIT]) and left (1.46 ± 0.05 ml [control], 1.41 ± 0.03 ml [sham], 1.3 ± 0.13 ml [LIET] and 1.21 ± 0.13 ml [HIIT]) testes.

### Microscopic findings

### Sperm parameters

We assessed sperm parameters of concentration, motility and morphology and compared them between the study groups. The mean sperm concentrations were 53.4 ± 3.98 10^6^ /ml (control), 52.11 ± 4.3 10^6^ /ml (sham), 57.1 ± 6.82 10^6^ /ml (LIET) and 54.7 ± 10.7 10^6^ /ml (HIIT). There were no significant differences in mean sperm concentration between the groups. We also did not observe any significant differences in terms of percentage of sperm motility between the control (24.8 ± 4.58%), sham (32.4 ± 7.13%), LIET (35.7 ± 2.7%) and HIIT (35 ± 4.24%) groups. However, the mean percentage of abnormal morphology was significantly (P<0.05) lower in the control (15.25 ± 1.09%) and LIET (17.37 ± 1.61%) groups compared to the sham (24.74 ± 0.78%) and HIIT (25.1 ± 2.21%) groups. The mean percentage of abnormal morphology was significantly lower (P<0.05) in the LIET group compared to the HIIT group (Table 2).

### Sperm lipid peroxidation

**Fig.2:**
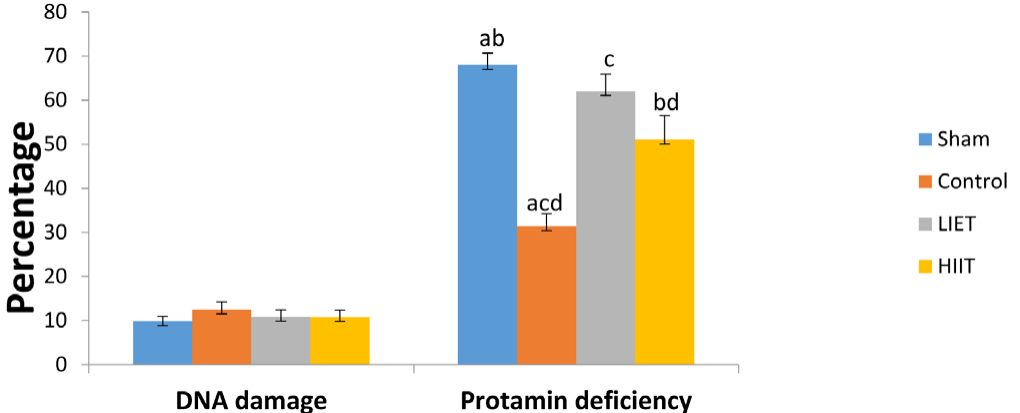
Comparison of the mean percentage of sperm lipid peroxidation (%) and intensity of sperm lipid peroxidation (IN) within the control, sham, HIIT and LIET groups. Common letters indicate significant differences between groups at P<0.05. HIIT; High-intensity interval training and LIET; Low-intensity endurance training.

Sperm lipid peroxidation was assessed by the BODIPY C11 probe and compared between the study groups (Fig.2). The mean percentages of sperm lipid peroxidation were significantly higher (P<0.05) in the LIET (34.2 ± 4.17%) and HIIT (33.11 ± 5.7%) groups compared to the sham (17.45 ± 1.72%) and control (17.6 ± 2.06%) groups, whereas the mean intensity of sperm lipid peroxidation did not significantly differ (P>0.05) between the sham (15.35 ± 0.3), control (19.83 ± 1.22), LIET (17.46 ± 1.05) and HIIT (22.23 ± 3.83) groups. 

**Table 2:** Comparison of body weight and sperm parameters within the study groups


Parameters	Groups			
	Sham	Control	LIET	HIIT

Body weight (g)	414.8 ± 4.2	452.5 ± 8.7	388.00 ± 9.37^a^	405.5 ± 10.09^a^
Sperm concentration (10^6^×ml)	52.11 ± 4.3	53.4 ± 3.98	57.1 ± 6.82	54.7 ± 10.7
Sperm motility (%)	32.4 ± 7.13	24.8 ± 4.58	35.7 ± 2.7	35 ± 4.24
Sperm abnormal morphology (%)	24.74 ± 0.78	15.25 ± 1.09^c^	17.37 ± 1.61^b^^c^	25.1 ± 2.21


Data are presented as mean ± SD.^ a^ ; Statistically significant difference between LIET and HIIT groups compared with the control and sham groups at P<0.05, ^b^; Statistically significant difference between the LIET group with the HIIT (P=0.04) and sham (P=0.03) groups at P<0.05, ^c^ ; Statistically significant difference between the LIET and control groups with the sham and HIIT groups at P<0.05, HIIT; High-intensity interval training, and LIET; Low-intensity endurance training.

### Sperm chromatin status

**Fig.3:**
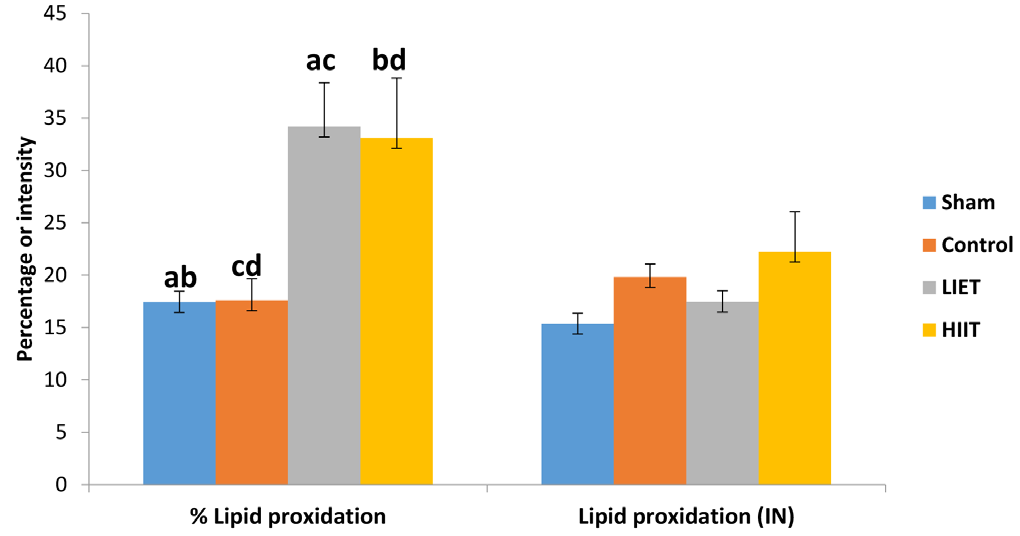
Comparison of the mean percentages of sperm DNA damage and protamine deficiency within the control, sham, HIIT and LIET groups. Common letters indicate significant differences between groups at P<0.05. HIIT; High-intensity interval training and LIET; Low-intensity endurance training.

Sperm DNA damage and protamine deficiency were assessed by AO and CMA3 staining, respectively, and compared between the study groups (Fig.3). We did not observe any significant differences in terms of percentage of sperm DNA damage between the sham (9.83 ± 1.07), control (12.5 ± 1.74), LIET (10.81 ± 1.6) and HIIT (10.75 ± 1.59) groups. However, the mean protamine deficiency in sperm was significantly higher in the sham (67.98 ± 2.7), LIET (62.04 ± 3.86) and HIIT (51.05 ± 5.48) groups compared to the control (31.44 ± 2.76) group. We also observed a significant difference between the sham and HIIT groups (P<0.05). 

## Discussion

Globalisation and changes in diet pattern have resulted in an increase in obesity, which appears to be a pandemic phenomenon. The impact of obesity on male reproductive function has been reported; in most cases, obesity could lead to reductions in the quality and quantity of sperm in men, oligozoospermia and azoospermia, and, subsequently, male infertility (23). Diet and/or exercise are introduced as an effective method for reducing obesity in humans and possibly improving semen quality parameters. Therefore, in this study we aimed to access the effects of LIET and HIIT exercise on sperm parameters and function in a male rat model of NAFLD.

The results of the current study showed that both LIET and HIIT exercise did not have any effect on the testicular weight and volume testicles in this NAFLD rat model. This result supported the findings of Edmonds et al. (24), who did not report any differences in the weights of the testicles of Zucker rats, which are a genetic model of obesity, compared to thin rats. Therefore, we concluded that dietinduced obesity in mice was not associated with a change in the average weight of the testicle. Interestingly, Joseph et al. (25) reported a 30% reduction in testicular weight in old (24-month-old) rats compared to young (6-monthold) rats, and treadmill exercise training significantly increased testicular weight in both young and old rats. Unlike the current study, Dominguez et al. (26) argued that endurance exercise training decreased testicular weight due to a reduction in relative oxygen pressure, and led to limited oxygen transport from the microvascular to testicle mitochondria and affected testicular function.

In the present study, both HIIT and LIET exercise had no effect on the main sperm parameters, including concentration and sperm motility, which differed from the results of other studies that suggested beneficial effects of exercise on gonadal fat, oxidative stress, adiposity index, sperm quality and fertility (27). In line with the latter study, Palmer et al. (28) demonstrated that both diet and exercise could improve sperm function in obese mice that were fed a high-fat diet. In addition, Yi et al. (29) reported that unlike both long-term moderate and high-volume exercises, only moderate-volume exercise improved the impacts of obesity on male reproductive function. Therefore, it could be concluded from these studies that the type and duration of exercise, as well as exercise with or without diet, could have different effects on sperm quality. In this regard, we previously showed that intervention of aerobic exercise and/or diet affected sperm concentration and motility in both obese and nonobese groups. This difference between the two studies could be related to the animal model (fatty liver vs. obese and non-obese), Type of exercise protocol (endurance and interval exercise vs. endurance exercise), adaptation time to the created condition (four months vs. three months), study animal (rat vs. mouse) and age (30). Based on a previous study, exercise by activating adaptive stress response pathways can increase cell resistance to stress and increase the expression of cytopathic protective proteins (e.g., thermal shock proteins), phase 2 enzymes [e.g., heme oxygenase-1 (HO-1)], and antioxidant enzymes (e.g., superoxide dismutase and HSP-72) content in the myocardium and other parts of the body (31). Therefore, exercise can partly lead to the activation of compensatory or adaptive mechanisms in the body during obesity. 

Unlike sperm concentration and motility, we observed a similar mean value for abnormal sperm morphology between the control and LIET groups in rats with fatty liver, whereas a high percentage of sperm with abnormal morphology were observed in the HIIT and sham groups. We concluded that high intensity training might have an adverse effect on sperm morphology, but did not change the integrity of sperm DNA. The mean sperm abnormal morphology was elevated in the control group. We first explained that the fatty liver model could affect spermatogenesis and increase the production of sperm with abnormal morphology. Unlike HIIT, LIET exercise could possibly improve the sperm morphology status. 

In this regard, Gomes et al. reported a decrease in activity of the enzyme sorbitol dehydrogenase (SDH) after HIIT. This enzyme converts sorbitol to fructose. Both spermatogonium and sperm use glucose and fructose as their main energy sources (32); therefore, we suggest that a deficiency in SDH could affect the spermatogenesis process following HIIT.

Unlike the means for sperm DNA damage, which were similar between the groups in the current study, the mean levels for sperm lipid peroxidation were significantly higher in both the LIET and HIIT groups compared to the control and sham groups. The intensity of lipid peroxidation in sperm were similar between the groups. This result supported the findings of Tartibian and Hajizadeh Maleki (33), who stated that long and intense periods of competitive sports like wrestling, boxing, judo, taekwondo and karate increase oxidative stress, cell damage, and disturb the balance of oxidants/ antioxidants. Nematollahi et al. (30) stated that unlike non-obese mice, aerobic exercise and/or diet (high or low fat) interventions increased the mean percentage of sperm lipid peroxidation in obese mice. According to the literature, exercise increases the consumption of oxygen in the skeletal muscles and leads to a significant increase in the production of oxidants (34). The plasma membrane of mammalian spermatozoa is rich in nonsaturated fatty acids and sperm is vulnerable to lipid peroxidation by oxidants. Based on the current study results, we suggest that the increase in lipid peroxidation in sperm from both exercise groups could not have a severe pathological effect on sperm parameters and DNA damage within the groups. In this regard, several studies have shown that exercise significantly decreased antioxidant activity such as superoxide dismutase and catalase in rat testis (35). Interestingly, Santos et al. reported impaired sperm function and oxidative stress in rat offspring of mothers fed an obesogenic diet. Exercise was effective in improving testicular oxidative stress, sperm antioxidant activity, sperm parameters and fertility in the rat offspring (27).

In this study, the mean value of sperm protamine deficiency was significantly higher in both exercise groups (LIET and HIIT) compared to the control group. This might account for an increased abnormality in the HIIT group and could be related to a high percentage of sperm lipid peroxidation in these groups (LIET and HIIT), which might hinder the histone/protamine exchange. Notably, despite the increased lipid peroxidation and protamine deficiency in both groups, there was only an increase in abnormal morphology in the HIIT group, and not in the LIET group. This was likely related to differences in exercise intensity. It is important to note that despite increased lipid peroxidation and protamine deficiency in both groups, the intensity of damage was not extensive enough to damage sperm DNA structiure which is always a time gap between oxidation and DNA fragmentation (36).

Subtle oxidative stress might not have a profound observable effect on semen parameters and chromatin integrity, but oxidants may target subtle regions of chromosomes. In this regard, a recent study has shown that promotors of genes involved in neurodevelopment related to behavioural characteristics like autism spectrum disorders, schizophrenia and bipolar disorder are more prone to oxidation and, thereby, DNA damage, which are not observable when assessing whole genomic integrity after exposure to oxidants. However, it may specifically target explicit regions of the genome (37). Thus, researchers believe that ageing and infertility increase the risk for Klinefelter syndrome (38). Any change in lifestyle that could lead to a subtle oxidative stress may have profound consequences on the health of the next generation. Therefore, measures should be taken when opting for fertility (37, 39). Considering the fact that we only observed subtle oxidative adverse effects of fatty liver and exercise, fertility care should be considered when planning for fertility. López-Lemus et al. (40) have demonstrated that NAFLD is a strongly associated factor with the severity of testicular epithelial damage. They suggested that weight reduction through diet and exercise in individuals with fatty liver disease would have positive effects on male fertility. 

Subtle oxidative stress might not have a profound observable effect on semen parameters and chromatin integrity, but oxidants may target subtle regions of chromosomes. In this regard, a recent study has shown that promotors of genes involved in neurodevelopment related to behavioural characteristics like autism spectrum disorders, schizophrenia and bipolar disorder are more prone to oxidation and, thereby, DNA damage, which are not observable when assessing whole genomic integrity after exposure to oxidants. However, it may specifically target explicit regions of the genome (37). Thus, researchers believe that ageing and infertility increase the risk for Klinefelter syndrome (38). Any change in lifestyle that could lead to a subtle oxidative stress may have profound consequences on the health of the next generation. Therefore, measures should be taken when opting for fertility (37, 39). Considering the fact that we only observed subtle oxidative adverse effects of fatty liver and exercise, fertility care should be considered when planning for fertility. López-Lemus et al. (40) have demonstrated that NAFLD is a strongly associated factor with the severity of testicular epithelial damage. They suggested that weight reduction through diet and exercise in individuals with fatty liver disease would have positive effects on male fertility. 

## Conclusion

The results of this study showed that both LIET and HIIT in the rat NAFLD model had no adverse effects on testicular morphometric parameters, sperm concentration, motility, and DNA integrity. However, the mean sperm lipid peroxidation and protamine deficiency were significantly higher in both exercise groups. Our study suggests that obese individuals with fatty liver who like to undergo exercise for weight reduction and overcome their fatty liver may postpone their fertility until full testicular adaptation is achieved by exercise or they may undergo antioxidant supplementation to minimise the adverse effects of oxidant by-products of exercise.
